# Inhaled sGC Modulator Can Lower PH in Patients With COPD Without Deteriorating Oxygenation

**DOI:** 10.1002/psp4.12308

**Published:** 2018-07-02

**Authors:** Sina Saffaran, Wenfei Wang, Anup Das, Walter Schmitt, Eva‐Maria Becker‐Pelster, Jonathan G. Hardman, Gerrit Weimann, Declan G. Bates

**Affiliations:** ^1^ School of Engineering University of Warwick Coventry West Midlands UK; ^2^ Clinical Science, Bayer Healthcare Barmen Germany; ^3^ School of Medicine University of Nottingham Nottingham UK

## Abstract

This study uses a highly fidelity computational simulator of pulmonary physiology to evaluate the impact of a soluble guanylate cyclase (sGC) modulator on gas exchange in patients with chronic obstructive pulmonary disease (COPD) and pulmonary hypertension (PH) as a complication. Three virtual patients with COPD were configured in the simulator based on clinical data. In agreement with previous clinical studies, modeling systemic application of an sGC modulator results in reduced partial pressure of oxygen (PaO_2_) and increased partial pressure of carbon dioxide (PaCO_2_) in arterial blood, if a drug‐induced reduction of pulmonary vascular resistance (PVR) equal to that observed experimentally is assumed. In contrast, for administration via dry powder inhalation (DPI), our simulations suggest that the treatment results in no deterioration in oxygenation. For patients under exercise, DPI administration lowers PH, whereas oxygenation is improved with respect to baseline values.

**Study Highlights**

**WHAT IS THE CURRENT KNOWLEDGE ON THE TOPIC?**

☑ A modulator of sGC has recently been shown in a clinical study to improve pulmonary hemodynamics in patients with COPD and PH as a complication. However, systemic administration of sGCs bears a high risk of deterioration of V/Q mismatch due to relief of hypoxic vasoconstriction.

**WHAT QUESTION DID THIS STUDY ADDRESS?**

☑ We aim to identify if administration of an sGC via DPI, instead of systemically, could reduce the negative impact of the drug on oxygenation.

**WHAT DOES THIS STUDY ADD TO OUR KNOWLEDGE?**

☑ Using a high‐fidelity pulmonary simulator, calibrated to data from three patients with COPD involved in a previous clinical trial, we showed that administering an sGC via DPI can reduce PH without deteriorating oxygenation, particularly when administration is combined with exercise.

**HOW MIGHT THIS CHANGE DRUG DISCOVERY, DEVELOPMENT, AND/OR THERAPEUTICS?**

☑ Our results highlight the potential advantages of administering sGCs to patients via DPI, rather than systemically.

Chronic obstructive pulmonary disease (COPD) is one of the leading causes of morbidity and mortality in most countries.^1^, ^2^ The World Health Organization estimates that COPD was the fifth leading cause of death in high‐income countries in 2001, and it was the sixth leading cause of death in nations of low and middle income.^3^ COPD has been identified as a major global health burden based on its high prevalence and significant healthcare costs.^4^, ^5^

A serious complication of COPD is pulmonary hypertension (PH), a progressive and debilitating condition associated with a sustained increase in mean pulmonary artery pressure (mPAP) that results from excessive vasoconstriction and remodeling of the pulmonary arteries.^6^ It is associated with shorter survival and has been seen as a predictive factor for worse clinical outcomes and frequent use of health resources.^7^, ^8^ Accordingly, there has been significant interest in exploring PH‐specific therapies in patients with COPD. Various vasodilating drugs with different modes of action have been investigated in clinical studies.^9^ Most of these studies considered systemic applications (oral administration), although some also considered administration via inhalation. Generally, these studies aimed for a dilation or relaxation of the pulmonary arterial vessels, thus lowering pulmonary vascular resistance. On the other hand, unselective vasodilation of pulmonary vessels may also lead to a relief of hypoxic vasoconstriction in low‐ventilating/non‐ventilating areas of the lung, and consequently to increased ventilation‐perfusion (V/Q) mismatch and a deterioration of oxygenation.

One of the most extensively investigated drugs in COPD‐PH is the phosphodiesterase‐5 (PDE‐5) inhibitor sildenafil.^10^, ^11^, ^12^, ^13^, ^14^, ^15^ When given systemically (i.e., oral or intravenous administration) in acute studies sildenafil consistently resulted in a reduction of mPAP and an improvement of exercise capacity and 6‐minute walking distance.^10^, ^12^, ^13^ Although, during exercise there seems to be no adverse reaction, at rest the hemodynamic changes occurred at the expense of worsening gas exchange due to increased V/Q mismatching.^13^ In chronic studies, no clear positive effect of the treatment with sildenafil, as compared to placebo, could be observed with positive^14^ as well as negative outcomes being reported.^11^, ^15^

Trials have also been undertaken with bosentan, an oral endothelin receptor antagonist, in treating PH in COPD. Stolz *et al*.^16^ found in patients with severe COPD that those treated with bosentan incurred a decreased quality of life, worsening arterial oxygen saturation, and an increased alveolar‐arterial gradient with no change in exercise capacity. In another study, the treatment group benefited from significant improvements in mPAP, pulmonary vascular resistance (PVR), and the 6‐minute walk distance without a significant decline in oxygenation.^17^

Inhalation therapy was investigated in patients with COPD‐PH with inhaled nitrogen oxide (iNO)^18^, ^19^, ^20^, ^21^, ^22^, ^23^, ^24^, ^25^ and the prostacyclin analogue iloprost.^26^, ^27^, ^28^ The trials with iNO consistently demonstrated a considerable and concentration‐dependent reduction of PVR.^18^, ^19^, ^20^, ^21^, ^22^, ^23^, ^25^ The response of gas exchange, in particular of oxygenation, to iNO therapy is, however, heterogeneous. Although, clear concentration dependence cannot be derived from the different studies, there is at least evidence that at lower iNO concentrations arterial oxygenation is improved or remains unchanged with iNO inhalation,^18^, ^21^, ^23^, ^25^ whereas at higher concentrations (>20 ppm) there is no gain in oxygenation^22^ or even a deterioration.^19^, ^20^ The latter response is presumably the result of increased V/Q mismatch caused by nitrogen oxide releasing hypoxic vasoconstriction in poorly ventilated regions of the lungs. However, the opposite effect was also observed in another study, in which an improvement of partial pressure of oxygen (PaO_2_) was recorded with high nitrogen oxide concentrations.^18^

An inconsistent picture also emerges from the studies with inhaled iloprost. Although Boeck and colleagues^26^ did not find positive effects but instead a worsening of gas exchange for two different iloprost doses in a cross‐over study, two other studies reported improvements in V/Q matching, gas exchange, and exercise tolerance.^27^, ^28^

Recently, riociguat, a stimulator of soluble guanylate synthase (sGC) has been approved for treatment of pulmonary arterial hypertension and chronic thromboembolic PH after it showed improved 6‐minute walk distance, compared with placebo, and also improved PVR, functional class, dyspnea, and health‐related quality of life in these diseases.^29^, ^30^ riociguat was also investigated in a single dose study with patients with COPD with borderline or manifest PH (mPAP ≥23 mmHg).^31^ Similar as for other therapies, significant reductions of mPAP and PVR could be demonstrated in this patient population. Although some reduction in oxygenation occurred with orally administered riociguat in these studies, this was not at levels that were judged to be clinically relevant.

Overall, the results from the different studies discussed above give rise to conclusions that:

- The pharmacological principle of vasodilation is generally appropriate for improving pulmonary hemodynamics and exercise tolerance of patients with COPD‐PH;
- Systemic administration of drugs bears a high risk of deterioration of V/Q mismatch due to relief of hypoxic vasoconstriction;
- Inhaled administration shows positive effects and may, in contrast to systemic administration, lead to improved V/Q mismatching, although also the opposite can happen if the distribution of the drug is not strictly limited to well ventilated regions of the lungs, or if alveolar absorption is high and considerable systemic exposure occurs after inhalation.

One potential limitation to consider is the fact that inhalation with a metered dose or dry powder inhalation (DPI), typically used in lung diseases, such as COPD and asthma,^32^, ^33^ is associated with a deep breath. This could, however, deteriorate the advantage of inhaled administration, as a deep breath may result in drug particles being deposited in lung areas that are not ventilated at rest.

The complexity of the findings summarized above highlights the fact that the role of different therapies and corresponding administration methods in COPD‐related PH needs further exploration. In this study, simulation approaches using a high‐fidelity simulation model were adopted to evaluate the effects of a vasodilator, in terms of hemodynamics and oxygenation, in patients with COPD with PH.

In the following, we demonstrate that the simulation model is able to recapitulate observed changes in gas exchange after systemic administration of the sGC stimulator riociguat, based on the experimentally determined reductions of PVR. Thus validated, the model is then used to evaluate the effects of alternative administration methods (DPI via a deep breath and inhalation via normal breathing; e.g., using a ventilator) of the drug. We also quantitatively investigate the consequences of administering the drug to patients while they are under exercise.

## METHODS

### Pulmonary simulator

The simulator used in this study is a multicompartmental computational model that uses an iterative technique to simulate integrated respiratory and cardiovascular pathophysiological scenarios.^34^, ^35^, ^36^, ^37^, ^38^ In contrast to previous models of COPD pathophysiology that included only two or three alveolar compartments,^39^ our model allows the user to define up to several hundred individual compartments (each with its own individual mechanical characteristics) to be implemented in the simulation. Each of these alveolar compartments has a unique and configurable bronchiolar resistance, PVR, stiffness index, and extrinsic pressure. The ability to adjust these parameters individually across all alveolar compartments allows the model to recreate the heterogeneous effects of COPD on the overall physiology of the lungs. The model also includes specific equations to represent the effects of alveolar collapse, threshold opening pressure, alveolar stiffening, and airway obstruction. The net effect of these components of the simulation is that the defining, clinical features of COPD may be observed in the model: alveolar gas trapping (with intrinsic positive end‐expiratory pressure), collapse‐reopening of alveoli (with gradual reabsorption of trapped gas if reopening does not occur), limitation of expiratory flow, and increased functional residual. A complete description of the simulator, including all underlying equations, is provided in the **Supplementary File**.

### Model matching to patient data

The model was matched to individual patient data by using a global optimization algorithm,^40^ as described in preceding publications,^35^, ^36^ and in the **Supplementary File**. For the present work, we matched the pulmonary simulator to the characteristics of three patients with COPD‐PH with differing gas exchange properties, which were included in the previous study with riociguat.^31^ The respective data on PaO_2_, partial pressure of carbon dioxide (PaCO_2_), dead space fraction, and V/Q at baseline (see **Table** 1) were taken from the study report. The three example datasets were chosen in order to cover a wide spectrum of COPD pathophysiology. For two of the patients, the multiple inert gas elimination technique was applied in order to determine data on V/Q mismatch, and, thus, for these patients we also present a comparison of data and model outputs on V/Q.

**Table 1:** Patient matching results

	Patient 1			Patient 2			Patient 3		
	Data	Model	Error	Data	Model	Error	Data	Model	Error
PaO_2_, mmHg	129.2	129.3	0.08%	76.8	76.69	0.14%	66.0	65.5	0.76%
PaCO_2_, mmHg	44.1	44.12	0.05%	49.8	49.85	0.10%	32.5	32.3	0.62%
Dead space, %	45.8	45.8	0.00%	39.9	39.8	0.25%	–	40.0	–
0.001<V/Q<0.1%	33.1	33.2	0.30%	13.6	13.61	0.07%	–	22.4	–
0.1<V/Q<10%	63.7	64.4	1.10%	84.9	84.29	0.72%	–	75.1	–

PaCO_2_, partial pressure of carbon dioxide; PaO_2_, partial pressure of oxygen; V/Q, ventilation‐perfusion.

**Table** 1 reports the matching results for all three patients with COPD considered. From **Table** 1, it is clear that the models are closely matched to the data with percentage errors below or around 1%. In order to assess the robustness of subsequent findings, 100 random parameter sets within ±5% of the best fit for each patient were also generated. All the analyses described below were applied to the best‐fit model, and findings were subsequently checked for consistency on all 100 parameter sets around each optimal patient parameter set.

### Modeling of drug effects and application methods

**Figure 1:**
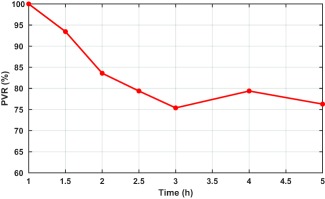
Mean change of pulmonary vascular resistance (PVR) from baseline over time in patients receiving a single dose of riociguat 2.5 mg.

The change from baseline of PVR after drug administration was used as an input for the model. In order to specifically simulate the behavior of riociguat, the respective mean relative PVR curve, as measured for a dose of 2.5 mg riociguat,^31^ was considered for the present simulations (see **Figure** 1). Individual data on PVR changes after treatment with riociguat were not published in ref. 31 and, therefore, the same mean profile was used for all simulated patients.

In the simulator, the pulmonary vessels are modeled as a parallel network with 100 compartments. Each compartment has a vascular resistance denoted as *PVR_i_*, and the total pulmonary vascular resistance is defined as:

(1) $${PVR}_{tot}=\;\frac{1}{\frac{1}{{PVR}_{1}}+\frac{1}{{PVR}_{2}}+\cdots +\frac{1}{{PVR}_{100}}}$$

Thus, reduction of PVR is captured by changing the resistances of individual compartments due to drug administration. For a given temporal profile of the drug‐induced change of PVR (see **Figure** 1), the temporal changes of *PVR_i_* in the individual compartments are calculated as follows.

After systemic application, the drug substance is delivered to the lungs through the blood circulation. The assumption in this case is that the drug will act equally on all compartments with blood going through them, and produce the same amount of vascular resistance reduction:

(2) $$\bar{PVR_{i}}\left(t\right)={PVR}_{i}^{baseline}-\alpha \left(t\right)\cdot \Delta_{PVR}$$

where $\Delta_{PVR}$ is a fixed value denoting the equal reduction in resistance for all compartments, and $\alpha \left(t\right)$ is a time‐dependent variable reflecting the variability of the drug effects over time. Combining Eq. 1 and Eq. 2, the desired PVR reduction shown in **Figure** 1 can be straightforwardly implemented by adjusting α.

In the case of inhaled application of an aerosol, for example, containing the drug, we assume that deposition of the drug in different compartments of the lungs is proportional to the extent of ventilation in these compartments. This means that the drug only reaches compartments of the lungs that are ventilated under normal breathing conditions. In addition, it is assumed that an inhaled application will not lead to systemic exposure causing any systemic effect, nor any effect in the parts of the lungs not directly addressed via inhalation.

Despite the assumption that the drug reaches only ventilated areas, and, thus, only in a part of the lungs will a vasodilatory effect be induced, we assume that a similar reduction of total PVR is achievable as observed with the systemic application (**Figure** 1). This assumption is supported by the fact that for inhaled iNO PVR reductions in the range of 25–30% were observed.^18^, ^22^, ^23^ Furthermore, it is assumed that the inhalation process is short, compared to the absorption and induction effect and, thus, the time course of the PVR reduction would be the same as the one observed after oral administration (**Figure** 1). Furthermore, it is assumed that the drug effect is proportional to the amount of drug deposited. Accordingly, the changes in vascular resistance due to treatment with the drug for each compartment is proportional to the ventilation reaching that compartment, and consequently the changed resistance $\bar{PVR_{i}}$ is given by:

(3) $$\bar{PVR_{i}}\left(t\right)={PVR}_{i}^{baseline}\left(1-\beta \left(t\right)\cdot \Delta_{PVRi}\right)$$

where $\Delta_{PVRi}=V_{Ti}/V_{T}$, $\beta \left(t\right)$ reflects the temporal effect of the drug, $V_{Ti}$ is the ventilation of alveolar compartment *i*, and $V_{T}$ is the total tidal volume. Combining Eq. 1 and Eq. 3, the desired PVR reduction in **Figure** 1 can be implemented straightforwardly by adjusting β.

Independent of the application device, DPI always needs a deep breath for inhaling the drug dose. Deep inhalation causes a rapid increase of lung volume, which results in dilating of the airway and a temporary reduction of airway resistance,^41^, ^42^ which will affect how the drug is delivered. We model the effect of this reduction of airway resistance, with the underlying assumption that compartments with larger initial volumes will exhibit smaller reductions in airway resistance. Given that total parallel airway resistance *R_aw_* is given by:

(4) $$R_{aw}=\frac{1}{\frac{1}{R_{aw1}}+\frac{1}{R_{aw2}}+\cdots +\frac{1}{R_{aw100}}}$$

the new airway resistance for compartment *i* is then given by:

(5) $${\bar{R}}_{\mathrm{awi}}=R_{\mathrm{awi}}\cdot \Delta_{\mathrm{awi}},\;where\;\Delta_{\mathrm{awi}}=\;\frac{1}{1+\frac{X_{aw}}{Vol_{i}}}$$

where *Vol_i_* is the volume of compartment *i*, and *X_aw_* is a scaling parameter related to tidal volume. It can be seen that a smaller *Vol_i_* will lead to a bigger reduction of airway resistance. For example, setting *X_aw_* = 20, we have that: (1) if *Vol_i_* = 20 mL, Δ_awi_ = 0.5; and (2) if *Vol_i_* = 80 mL, Δ_awi_ = 0.8. The deep inhalation is replicated in the virtual patients by applying a threefold increase in tidal volume for a period of five respiratory cycles.

**Figure 2:**
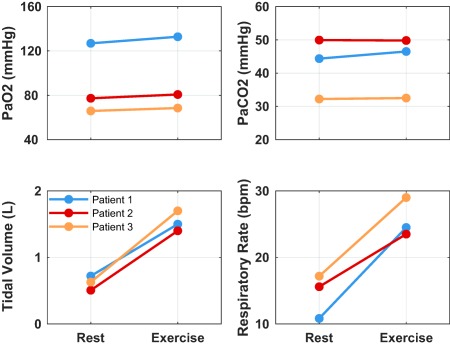
Values for tidal volume and respiratory rate as well as the corresponding changes to partial pressure of oxygen (PaO_2_) and partial pressure of carbon dioxide (PaCO_2_) at rest and under exercise.

In patients with COPD, oxygen consumption (VO_2_) is more restrained by impaired pulmonary ventilation than by oxygen delivery, which imposes exercise limitations upon these patients. Under exercise, VO_2_ for patients with COPD has been shown to increase to 0.7 L.min^−1^ (SD = 0.25), on average.^43^, ^44^ In response to the elevated VO_2_, minute ventilation and cardiac output rise accordingly to deliver more oxygen to the tissues.^44^, ^45^ In our model, the physiological effects of initiating exercise were simulated by progressively increasing VO_2_ every minute to 0.35, 0.5, 0.6, 0.65, and 0.7 L.min^−1^ in the virtual patients. Minute ventilation was subsequently increased as required to maintain the arterial blood gases at their pre‐exercise values, using the exponential relation between tidal volume and respiratory rate in ref. 46. Cardiac output was raised to 8.2 L.min^−1^ according to the average change observed in ref. 44, and following the increase in cardiac output an additional 10% reduction in PVR to that caused by the drug was applied.^46^ Exercise was simulated to start 30 minutes after administration of the drug by inhalation with deep breath and continues for 90 minutes. The at‐rest and under‐exercise values for tidal volume and respiratory rate as well as the corresponding changes to PaO_2_ and PaCO_2_ due to exercise are presented in **Figure** 2.

## RESULTS

### Systemic application

**Figure 3:**
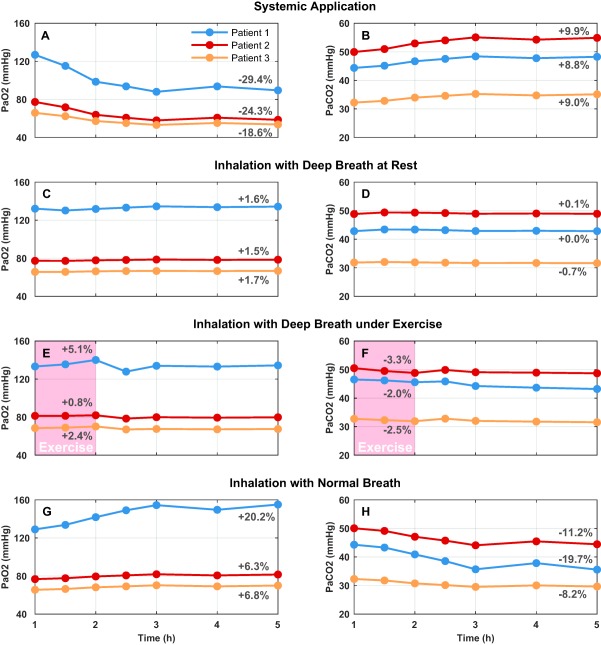
Simulation results for patients using different drug administration methods. PaCO_2_, partial pressure of carbon dioxide; PaO_2_, partial pressure of oxygen.

The average PVR reduction profile observed with 2.5 mg riociguat was applied to the three configured virtual patients with COPD for each of the three application methods (systemic, inhaled, and DPI). The changes in gas exchange parameters, PaO_2_, and PaCO_2_ over time were then recorded, and are shown for each patient in **Figure** 3 **a,b**. In the case of systemic application, reductions in PaO_2_ and increases in PaCO_2_ were observed for all three patients. For systemic application, the average percentage change for PaO_2_ is −24.1% and for PaCO_2_ is +9.2%, which are consistent with results reported in a previous clinical study with riociguat.^31^

### Inhaled application

In contrast to the systemic application, an inhaled administration leads to considerable improvements in oxygenation, as shown in **Figure** 3 **g,h**. The maximum increases of PaO_2_ are 20.2%, 6.3%, and 6.8% with an average of 11.1%, whereas the maximum decreases of PaCO_2_ are −19.7%, −11.2%, and −8.2%, respectively, with an average of −13.0%. This is as expected, because, in this case, no deep breaths are required on the part of the patients and, thus, the entire compound is delivered only to the normally ventilated regions of the lungs.

### Dry powder inhaler application at rest and under exercise

**Figure** 3 **c,d** shows the effects of application of the same compound using DPI via a deep breath at rest, which is a more realistic scenario for an inhaled therapy in COPD. With the same PVR reduction profile, changes of blood gas values are in between those calculated for the systemic and continuous inhaled applications. In fact, it can be seen that PaO_2_ is slightly increased and PaCO_2_ is slightly reduced for all three patients. The maximum increases of PaO_2_ are 1.6%, 1.5%, and 1.7%, respectively, with an average of 1.6%. The maximum changes of PaCO_2_ are 0.0%, 0.1%, and −0.7%, respectively, with an average of −0.2%. **Figure** 3 **e,f** shows that undertaking exercise for 1 hour after application by DPI produces further improvements in oxygenation, with maximum increases of PaO_2_ of 5.1%, 0.8%, and 2.4% with an average of 2.8%, and maximum decreases of PaCO_2_ of −2%, −3.3%, and −2.5%, respectively, with an average of −2.6%.

### Robustness analysis

**Figure 4:**
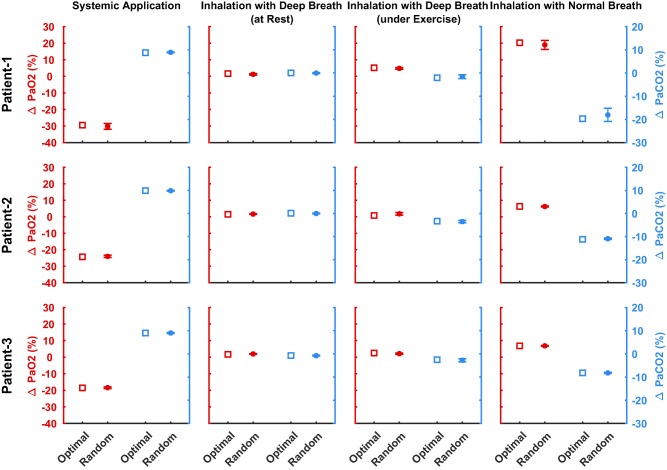
Comparison of the maximum change in partial pressure of oxygen (PaO_2_) and partial pressure of carbon dioxide (PaCO_2_) observed for each patient and each application method using the optimal parameter set (squares) with the average maximum change in PaO_2_ and PaCO_2_ calculated using 100 random parameter sets (circles with one SD as error bars).

To test the robustness of our results, for each patient, all simulations (i.e., systemic application, inhalation with deep breath at rest and under exercise, and inhalation with normal breath) were repeated on 100 parameter sets randomly chosen within bounds of ±5% of the optimal parameter set found by means of global optimization. **Figure** 4 compares the maximum change in PaO_2_ and PaCO_2_ observed for each patient and each application method using the optimal parameter set (squares) with the average maximum change in PaO_2_ and PaCO_2_ calculated using 100 random parameter sets (circles with one SD as error bars). The outcomes confirm the consistency of the observed responses to the different methods of drug administrations (i.e., the average maximum change from baseline) for PaO_2_ and PaCO_2_ across all random sets for each patient closely matches the maximum changes reported when using the optimal parameter set during our previous analysis. **Supplementary Figures S3–S5** show time‐response plots for the performed robustness analysis.

## DISCUSSION

This is the first study to investigate the efficacy of sGC modulators in patients with COPD with PH by using computer simulation. The capability of the simulator to accurately describe the pathophysiological characteristics of gas exchange in patients with COPD has already been demonstrated in a previous study.^36^ That study showed that by including sufficient numbers of alveolar compartments in the model, accurate representations of both steady‐state blood gases and ventilation‐perfusion mismatch via V/Q curves could be obtained. In this study, we further demonstrated close matching of the simulator to data on three patients whose hemodynamics was invasively monitored in the study reported by Ghofrani *et al*.^31^ Calculations of blood gas concentrations for these virtual patients, considering the observed temporal profile of average PVR changes after oral administration of riociguat, resulted in predictions of changes in O_2_ and CO_2_ partial pressures that were consistent with those observed in the previous clinical study. These results can be considered as a strong validation of the capability of the pulmonary simulator to reliably describe the effects on gas exchange of compounds acting on the vascular resistance, in particular those stimulating the sGC activity.

As already discussed in previous publications, the systemic application of vasodilating drugs may lead to a worsening of V/Q mismatch and in consequence to an impairment of blood gas concentrations, which limits their clinical use. This arises due to the nonselective distribution of the drug to all parts of the lungs, which together with inhibition of hypoxic pulmonary vasoconstriction leads to an excess of blood flowing to poorly ventilated parts of the lungs.

One potential way to avoid increases of V/Q mismatch is via inhaled administration, as long as the drug can be expected to only reach the ventilated parts of the lungs. This avoids increasing the perfusion of nonventilated lung compartments, provided systemic exposure stays low enough after inhalation not to be effective. Our results from simulations based on this scenario with inhalation of a hypothetical sGC modulating drug, which can be applied to act on the lung selectively, supports this hypothesis when it is administered by normal breathing. In contrast to the findings from systemic administration, the inhaled application led to improved oxygenation, because perfusion of ventilated lung compartments was improved and effects on less well/nonventilated areas were limited. However, if the drug is formulated as a dry powder, its inhalation will usually be connected with a deep breath, which could cause a certain proportion of the compound to be deposited in regions of the lungs that are not, or poorly, ventilated when returning to normal breathing at rest. To quantitatively investigate the trade‐offs involved, we also modeled the effect of inhaled administration of a compound inducing the same effect on total PVR as 2.5 mg oral riociguat in COPD‐PH with a deep breath, causing the temporary recruitment of less well ventilated areas. The resulting simulations reveal that, although administering the drug by a deep breath may not improve oxygenation to the same extent as inhalation with normal breathing, it does avoid the potential deterioration in gas exchange associated with systemic drug administration. Moreover, when administered under exercise, most of the non/poorly ventilated parts of the lungs in which the drug is deposited due to deep breathing become ventilated again as a result of increased minute ventilation, leading to further improvements in oxygenation. Interestingly, these findings are also in agreement with the results of a clinical trial on the systemic administration of the PDE‐5 inhibitor sildenafil, which produced a worsening of gas exchange due to increased V/Q mismatching at rest, but not under exercise.^13^

A robust analysis of these results performed by means of randomly selecting 100 model parameter sets around the optimal values for each patient produced results that were uniformly consistent with the above findings. Our results highlight the potential advantages of administering sGCs to patients via DPI, rather than systemically, particularly when drug administration is combined with exercise.

## Conflict of Interest

The authors declared no competing interests for this work.

## Source of Funding

This work was supported by Bayer Healthcare via a research grant to the University of Warwick, and by EPSRC via Research Grant EP/P023444/1.

## Author Contributions

S.S., W.W., A.D., W.S., E.M.B., G.W., J.G.H., and D.G.B. wrote the manuscript. W.S., E.M.B., G.W., and D.G.B. designed the research. S.S., W.W., and A.D. performed the research. S.S., W.W., A.D., W.S., E.M.B., G.W., J.G.H., and D.G.B. analyzed the data.

## Supporting information

An inhaled sGC modulator can lower PH in COPD patients without deteriorating oxygenation (Supplementary File).
