# Supplementary material for: Inhaled sGC Modulator Can Lower PH in Patients With COPD Without Deteriorating Oxygenation
Source: CPT Pharmacometrics Syst Pharmacol. 2018 Jul 2;7(8):491–8. doi: 10.1002/psp4.12308 (PMC6118299; doi:10.1002/psp4.12308)
Supplement: Supplementary file 1 — An inhaled sGC modulator can lower PH in COPD patients without deteriorating oxygenation (Supplementary File). [file PSP4-7-491-s001.docx]

**An inhaled sGC modulator can lower PH in COPD patients without deteriorating oxygenation (Supplementary File)**

Sina Saffaran^1^, Wenfei Wang^1^, Anup Das^1^, Walter Schmitt^2^, Eva-Maria Becker-Pelster^2^, Jonathan G. Hardman^3^, Gerrit Weimann^2^ and Declan G. Bates^1*^

Author affiliations: School of Engineering, University of Warwick, UK, ^2^Clinical Science, Bayer Healthcare, Germany, ^3^School of Medicine, University of Nottingham, UK.

^*^Corresponding author: Declan G. Bates (PhD), School of Engineering, University of Warwick, Coventry CV4 7AL, UK, ph: +44(0)7876876952, [d.bates@warwick.ac.uk](mailto:d.bates@warwick.ac.uk)

**Supplementary Material**

**SECTION A: Simulation Model Description and Equations**

The model employed in this paper has been developed over the past several years and has been applied and validated on a number of different studies (1-8). The model is organized as a system of several components, each component representing different sections of pulmonary dynamics and blood gas transport, e.g. the transport of air in the mouth, the tidal flow in the airways, the gas exchange in the alveolar compartments and their corresponding capillary compartment, the flow of blood in the arteries, the veins, the cardiovascular compartment, and the gas exchange process in the peripheral tissue compartments. Each component is described as several mass conserving functions and solved as algebraic equations, obtained or approximated from the published literature, experimental data and clinical observations. These equations are solved in series in an iterative manner, so that solving one equation at current time instant $(t_{k})$ determines the values of the independent variables in the next equation. At the end of the iteration, the results of the solution of the final equations determine the independent variables of the first equation for the next iteration.

Figure S1.Diagrammatic representation of the model and its main features.


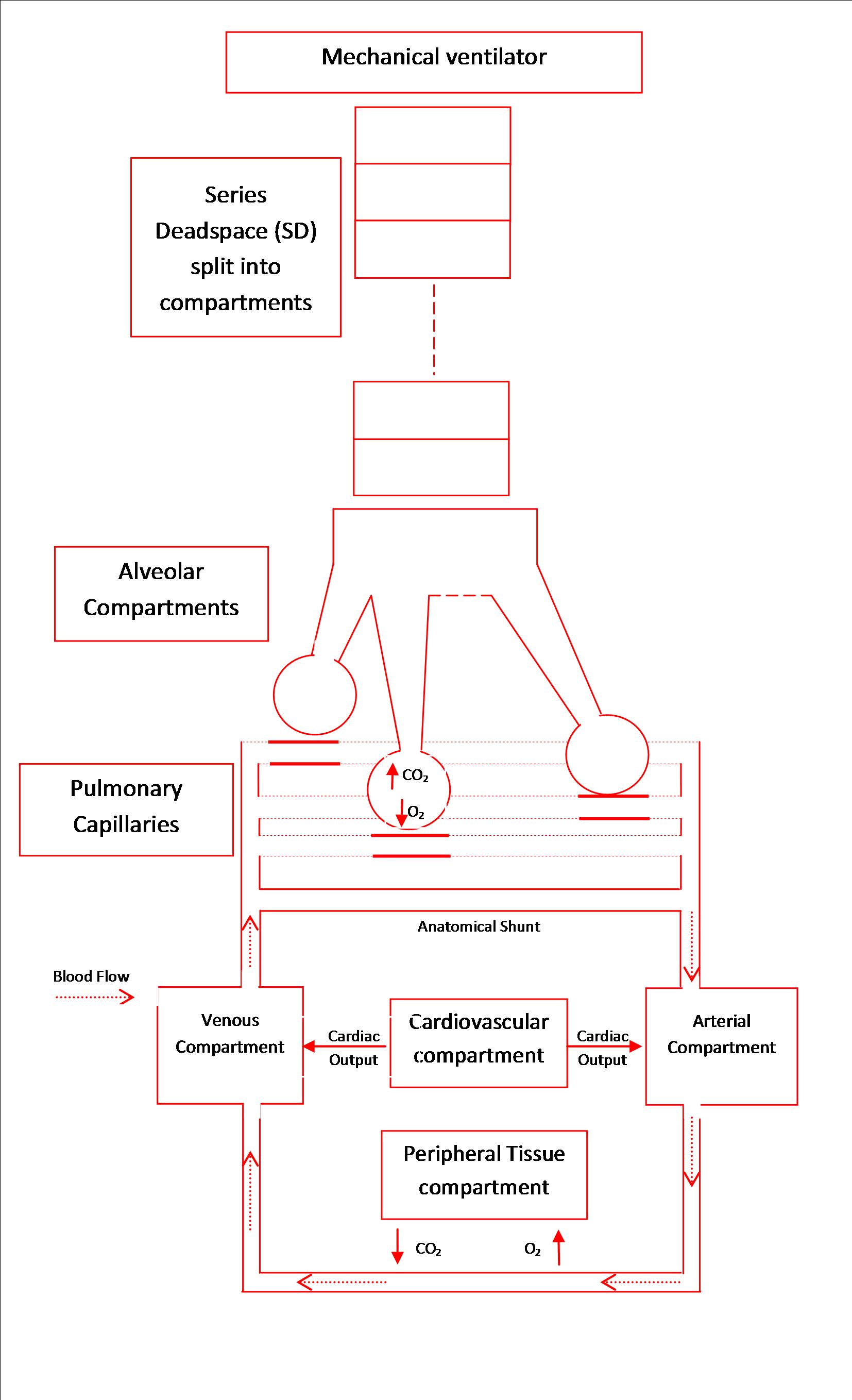

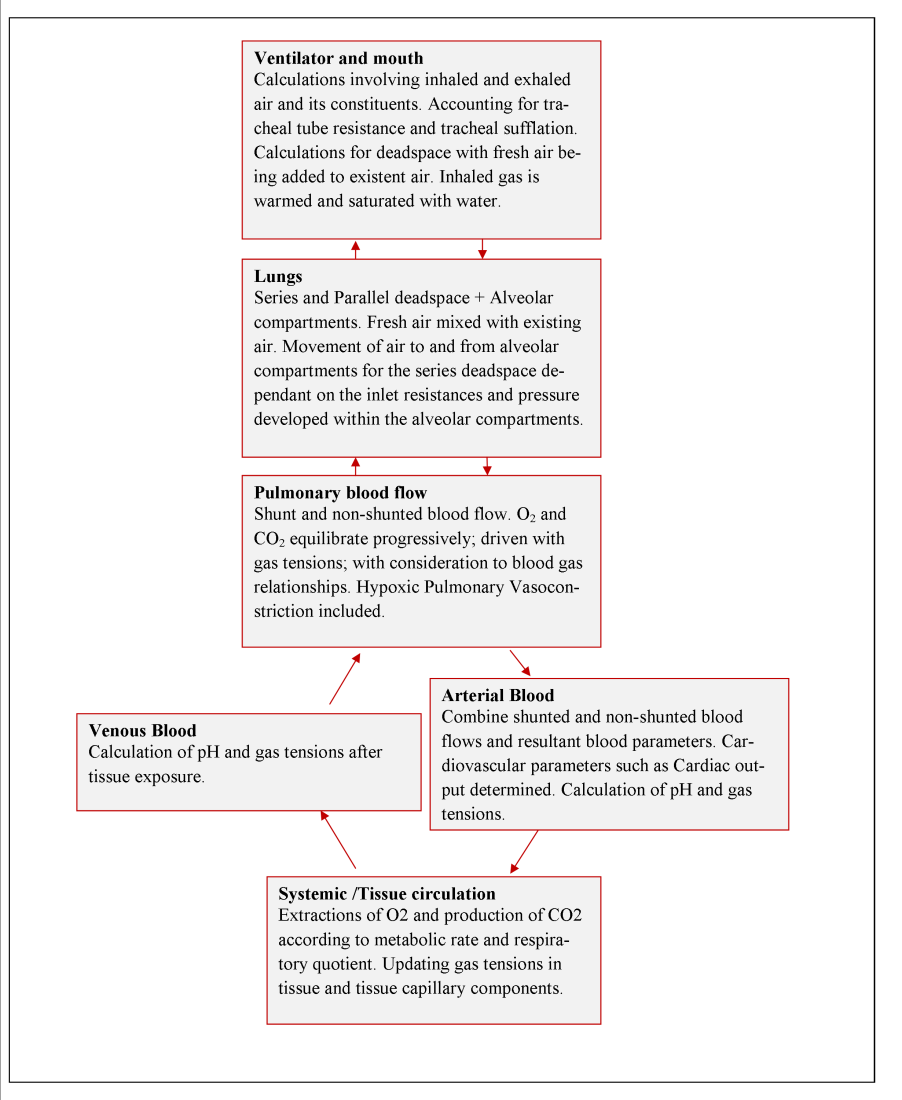


The iterative process continues for a predetermined time, *T*, representing the total simulation time, with each iteration representing a ‘time slice’ *t* of real physiological time (set to 30 ms). At the first iteration$(t_{k}, k=0)$, an initial set of independent variables are chosen based on values selected by the user. The user can alter these initial variables to investigate the response of the model or to simulate different pathophysiological conditions. Subsequent iterations ($t_{k}= t_{k-1}+t$) update the model parameters based on the equations below.

The pulmonary model consists of the mechanical ventilation equipment, anatomical and alveolar deadspace, anatomical and alveolar shunts, ventilated alveolar compartments and corresponding perfused capillary compartments. The pressure differential created by the mechanical ventilator drives the flow of gas through the system. The series deadspace (SD) is located between the mouth and the alveolar compartments and consists of the trachea, bronchi and the bronchioles where no gas exchange occurs. Inhaled gases pass through the SD during inspiration and alveolar gases pass through the SD during expiration. In the model, an SD of volume 60ml is split into 50 stacked layers of equal volumes ($N_{SD}$ = 50). No mixing between the compartments of the SD is assumed.

Any residual alveolar air in the SD at the end of expiration is re-inhaled as inspiration is initiated. This residual air is composed of gases exhaled from both perfused alveolar compartments (normal perfusion) and the parallel deadspace (PD) (alveolar compartments with limited perfusion). Therefore, the size of deadspace (SD and PD) can have a significant effect on the gas composition of the alveolar compartments.

The inhaled air is initially assumed to consist of five gases: oxygen (O_2_), nitrogen (N_2_), carbon dioxide (CO_2_), water vapour (H_2_O) and a 5th gas (α) used to model additives such as helium or other anaesthetic gases. During an iteration of the model, the flow (*f*) of air to or from an alveolar compartment *i* at time $t_{k}$ is determined by the following equation:

$f_{i}(t_{k})=\frac{\left( p_{v}(t_{k})- p_{i}\left( t_{k} \right) \right)}{\left( \text{R}_{\text{u}}+\text{R}_{\text{A, }i} \right)}$ $\mathrm{for} i=1,\ldots,N_{A}$ [1]

where $p_{v}(t_{k})$ is the pressure supplied by the mechanical ventilator at $(t_{k})$, $p_{i}\left( t_{k} \right)$ is the pressure in the alveolar compartment $i$ at $(t_{k})$, R_u_ is the constant upper airway resistance and R_A,_ *_i_* is the bronchial inlet resistances of the alveolar compartment $i$. $N_{A}$is the total number of alveolar compartments (for the results in this paper, $N_{A}$ = 100). The total flow of air entering the SD at time $t_{k}$ is calculated by

$f_{SD}(t_{k}) = \sum_{i=1}^{N_{A}} f_{i}(t_{k})$ [2]

During the inhaling phase, then$f_{SD}\geq0$, while in the exhaling phase$f_{SD}<0$.

During gas movement in the SD, the fractions of gases in the layer $l$ of the SD,$F_{l,} (l =1,\ldots, N_{SD}$) is updated based on the composition of the total flow,$f_{SD}$, and the current composition of$F_{l,}$. If $f_{SD}$ ≥ 0, then air starts filling from the top layer ($l$ = 1) to the bottom layer ($l= N_{SD}$); and vice versa for $f_{SD}$ < 0.

The volume of gas $\text{x}$, in the $i^{th}$ alveolar compartment ($\text{v}_{i\text{, x}}$), is given by:

$v_{i, x}(t_{k}) = \left\{ \begin{aligned} v_{i, x}\left( t_{k-1} \right)-f_{i}(t_{k})\cdot\frac{v_{i, x}(t_{k-1})}{v_{i}{(t}_{k})} Exhaling \\ v_{i, x}(t_{k-1}) +f_{i}(t_{k}) \cdot F_{N_{SD}}(t_{k}) Inhaling \end{aligned} \right.$ $\mathrm{for} i=1,\ldots,N_{A}$ [3]

In [3], x is any of the five gases (O_2_, N_2_, CO_2_, H_2_O or α). The total volume of the $i^{th}$ alveolar compartment, $v_{i}$ is the sum of the volume of the five gases in the compartment.

$v_{i}(t_{k})= v_{i,O2}(t_{k})+ v_{i,N2}(t_{k})+v_{i,CO2}(t_{k})+v_{i,H2O}(t_{k})+v_{i,\alpha}(t_{k})$ [4]

For the alveolar compartments, the tension at the centre of the alveolus and at the alveolar capillary border is assumed to be equal. The respiratory system has an intrinsic response to low oxygen levels in blood which is to restrict the blood flow in the pulmonary blood vessels, known as Hypoxic Pulmonary Vasoconstriction (HPV). This is modelled as a simple function, resembling the stimulus response curve suggested by Marshall (9), and is incorporated into the simulator to gradually constrict the blood vessels as a response to low alveolar oxygen tension. The atmospheric pressure is fixed at 101.3kPa and the body temperature is fixed at 37.2°C.

At each $t_{k}$, equilibration between the alveolar compartment and the corresponding capillary compartment is achieved iteratively by moving small volumes of each gas between the compartments until the partial pressures of these gases differ by <1% across the alveolar-capillary boundary. The process includes the nonlinear movement of O_2_ and CO_2_ across the alveolar capillary membrane during equilibration.

In blood, the total O_2_ content (C_O2_) is carried in two forms, as a solution and as oxyhaemoglobin (saturated haemoglobin):

$\text{C}_{\text{O2}}(t_{k})=\text{S}_{\text{O2}}(t_{k-1})\cdot Huf\cdot\text{Hb + }\text{P}_{\text{O2}}(t_{k-1})\cdot O_{2sol}$ [5]

In this equation, S_O2_ is the hemoglobin saturation, *Huf* is the Hufner constant, $\text{Hb}$ is the hemoglobin content and $O_{2sol}$ is the O_2_ solubility constant. The following pressure-saturation relation, as suggested by (10) to describe the O_2_ dissociation curve, is used in this model:

$\text{S}_{\text{O2}}(t_{k}) =\left( \left( \left( \text{P}_{\text{O2}}^{3}(t_{k-1})+150\cdot\text{P}_{\text{O2}}(t_{k-1}) \right)^{-1}\times23400 \right)+1 \right)^{-1}$ [6]

$\text{S}_{\text{O2}}$ is the saturation of the hemoglobin in blood and $\text{P}_{\text{O2}}$ is the partial pressure of oxygen in the blood. As suggested by (11), $\text{P}_{\text{O2}}$has been determined with appropriate correction factors in base excess BE, temperature T and pH (7.5005168 = pressure conversion factor from kPa to torr):

$\text{P}_{\text{O2}}(t_{k})=7.5006\text{168 ∙ }\text{P}_{\text{O2}}(t_{k-1}) \cdot{10}^{\left[ 0.48\left( \text{pH}(t_{k-1})\text{-7.4} \right)-0.024\left( \text{T-37} \right)-0.0013\cdot\text{BE} \right]}$ [7]

The CO_2_ content of the blood (C_CO2_) is deduced from the plasma CO_2_ content (C_CO2_plasma) (12) by the following equation:

$\text{C}_{\text{CO2}}(t_{k})= \text{C}_{\text{CO2plasma}}(t_{k-1}) \cdot\left[ 1-\frac{0.0289\cdot\text{Hb}}{\left( 3.352-0.456 . \text{S}_{\text{O2}}(t_{k}) \right)\cdot\left( 8.142-\text{pH}(t_{k-1}) \right)} \right]$ [8]

where $\text{S}_{\text{O2}}$ is the O_2_ saturation, $\text{Hb}$ is the hemoglobin concentration and pH is the blood pH level. The coefficients were determined as a standardized solution to the McHardy version of Visser’s equation (13) by iteratively finding the best fit values to a given set of clinical data. The value of $\text{C}_{\text{CO2plasma}}$ is deduced using the Henderson-Hasselbach logarithmic equation for plasma C_CO2_ (14)

$\text{C}_{\text{CO2plasma}}{(t}_{k})=2.226\cdot s_{CO2} \cdot\text{P}_{\text{CO2}} (t_{k-1}) \left( 1+ {10}^{\left( \text{pH}(t_{k-1}) \text{ – pK'} \right)} \right)$ [9]

where $\text{s}$ is the plasma CO_2_ solubility coefficient and $\text{pK'}$ is the apparent pK (acid dissociation constant of the CO_2_ bicarbonate relationship). $\text{P}_{\text{CO2}}$ is the partial pressure of CO_2_ in plasma and ‘2.226’ refers to the conversion factor from miliMoles per liter to ml/100ml. (14) gives the equations for $s_{CO2}$ and $\text{pK'}$ as:

$s_{CO2} \text{= 0.0307 + 0.0057 ∙ }\left( 37-\text{T} \right)\text{ + 0.00002}{\cdot\left( 37-\text{T} \right)}^{2}$ [10]

$\text{pK' = 6.086 +0.042 ∙ (7.4 - pH}(t_{k-1}) \text{) + }\left( 38-\text{T} \right) \cdot\left( 0.00472+\left( 0.00139- \left( 7.4-\text{pH}(t_{k-1}) \right) \right) \right)$ [11]

$\text{P}_{\text{CO2}} \left( t_{k} \right)$is determined by incorporating standard Henry’s law and the $s_{CO2}$(the CO_2_ solubility coefficient above). For pH calculation, the Henderson Hasselbach and the Van Slyke equation (15) are combined. Below is the derivation of the relevant equation. The Henderson-Hasselbach equation (governed by the mass action equation (acid dissociation)) states that:

$\text{pH = pK + log }\left( \frac{bicarbonate concentration}{carbonic acid concentration} \right)$ [12]

Substituting pK=6.1 (under normal conditions) and the denominator $(0.225 \cdot\text{P}_{\text{CO2}})$ (acid concentration being a function of CO_2_ solubility constant 0.225 and P_CO2_ (in kPa)) gives:

$\text{pH}{(t}_{k})\text{ = 6.1 + }\log\left( \frac{\text{HCO}_{\text{3}}(t_{k-1})}{0.225 \cdot\text{P}_{\text{CO2}(t_{k})}} \right)$ [13]

For a given pH, base excess (BE), and hemoglobin content (Hb), HCO_3_ is calculated using the Van-Slyke equation as given by (15):

$\text{HCO}_{\text{3}}{(t}_{k})\text{ }= \left( \left( 2.3 \times\text{Hb}+7.7 \right)\times\left( \text{pH}(t_{k})-7.4 \right) \right)+ \frac{\text{BE}}{\left( 1-0.023 \times\text{Hb} \right)}+ 24.4$ [14]

The capillary blood is mixed with arterial blood using the equation below which considers the anatomical shunt ($Sh)$ with the venous blood content of gas x ($\text{C}_{\text{v, x}})$, the non-shunted blood content from the pulmonary capillaries ($\text{C}_{\text{cap, x}}$), arterial blood content $\text{(}\text{C}_{\text{a, x}})$, the arterial volume $\text{(v}_{a})$ and the cardiac output (CO).

$\text{C}_{\text{a, x}}(t_{k})=\text{ }\frac{\text{CO}(t_{k})\text{ ∙ }\left( Sh \cdot\text{C}_{\text{v, x}}(t_{k}) + \left( 1-Sh \right) \cdot\text{C}_{\text{cap, x}}(t_{k}) \right)+ \text{C}_{\text{a, x}}(t_{k})\cdot\left( \text{v}_{a}(t_{k})- \text{CO}(t_{k}) \right)}{\text{v}_{a}(t_{k})}$ [15]

The peripheral tissue model consists of a single tissue compartment, acting between the peripheral capillary and the *active* tissue (undergoing respiration to produce energy). The consumed O_2_ (V_O2_) is removed and the produced CO_2_ (V_CO2_) is added to this tissue compartment. Similarly to alveolar equilibration, peripheral capillary gas partial pressures reach equilibrium with the tissue compartment partial pressures, with respect to the nonlinear movement of O_2_ and CO_2_. Metabolic production of acids, other than carbonic acid via CO_2_ production, is not modelled. After peripheral tissue equilibration of gases, the venous calculations of partial pressures, concentrations and pH calculations are done using comparable equations as above.

A simple equation of renal compensation for acid base disturbance is incorporated. The base excess (BE) of blood under normal conditions is zero. BE increases by 0.1 per time slice if pH falls below 7.36 (to compensate for acidosis) and decreases by 0.1 per time slice if pH rises above 7.4 (under alkalosis). The shunt fraction ($Q_{S}/Q_{T}$) in the model is calculated as:

$Q_{S}/Q_{T} = (C_{cO2}{(t}_{k})-C_{aO2}{(t}_{k}))/(C_{cO2}{(t}_{k})-C_{vO2}{(t}_{k}) )$ [16]

where the end capillary oxygen content ($C_{cO2}$), the arterial oxygen content ($C_{aO2}$) and the mixed venous oxygen ($C_{vO2}$) content can also be obtained from the model. The total compliance ($E_{\mathrm{dyn}}$) of the lung in the model is calculated using the standard equation:

$E_{\mathrm{dyn}}{(t}_{k}) = (V_{\max}{(t}_{k})-V_{\min}{(t}_{k}))/(P_{\max}{(t}_{k})-P_{\min}{(t}_{k}))$ [17]

where the End-Inspiratory Lung Volume ($V_{\max}$), End-Expiratory Lung Volume ($V_{\min}$), maximum pressure in lung ($P_{\max}$) and the minimum pressure in lung ($P_{\min}$) are obtained directly from the model at the end of every breath.

The simulated patient is assumed to be under complete mechanical ventilation. Consequently, the effects of ventilatory autoregulation by the patient have not been incorporated into the models.

Each alveolar compartment has a unique and configurable alveolar compliance, alveolar inlet resistance, vascular resistance, extrinsic (interstitial) pressure and threshold opening pressure. For the $i$^th^ compartment of *N* alveolar compartments, the pressure $p_{i}$ is determined by:

$p_{i}{(t}_{k})=\left\{ \begin{aligned} S_{i}\left( v_{i}{(t}_{k})-V_{c} \right)^{2}-P_{ext,i} v_{i}{(t}_{k})>0 \\ 0 \end{aligned} \right. \mathrm{for} i=1,\ldots,N_{A}$ [18]

where

$S_{i}=k_{i}{N_{A}}^{2}/200000$ and $V_{c}=0.2V_{FRC}/N_{A}$

Equation [18] determines the alveolar pressure $p_{i}$ (as the pressure above atmospheric in cmH_2_O) for the $i$^th^ compartment of *N* number of alveolar compartments for the given volume of alveolar compartment, $v_{i}(t)$ in milliliters. The alveolar compartments are arranged in parallel and interact with the series deadspace with respect to the movement of gases. The flow of air into the alveolar compartments is achieved by a positive pressure provided by the ventilator and the air moves along the pressure gradient. The equation models the behavior of the intact lung / chest-wall complex. The use of the square of the difference between $v_{i}$ and *Vc* causes alveolar pressure to increase at volumes below *Vc*, leading to exhalation and a tendency to “snap shut” (mathematical note: the pressure with respect to volume is thus a U-shaped curve). $P_{ext}$ (per alveolar unit, in cmH_2_O) represents the *effective net pressure* generated by the sum of the effects of factors *outside each alveolus* that act to distend that alveolus; positive components include the outward pull of the chest wall, and negative effects include the compressive effect of interstitial fluid in the alveolar wall. Incorporating $P_{ext}$ in the model allows us to replicate the situation of alveolar units that have less structural support or that have interstitial oedema, and thus have a greater tendency to collapse. A negative value of $P_{ext}$ indicates a scenario where there is compression from outside the alveolus causing collapse. The parameter $S_{i}$ is a scalar that determines the intra-alveolar pressure for a given volume (with respect to a constant collapsing volume$V_{c}$) and is dependent on the parameter$k$. The units of $S_{i}$ are cmH_2_O ml^-2^. Finally, $V_{c}$ is defined as a “constant collapsing volume” at which the alveolus tends to empty (through Laplace effects) and represents a fundamental mechanical property of tissue and surfactant. $V_{FRC}$ is the resting volume of the lung (assumed to be 3 litres).

The effect of the three parameters on the volume–pressure relationship of the alveolar compartments can be observed in the following Figure S2.

| 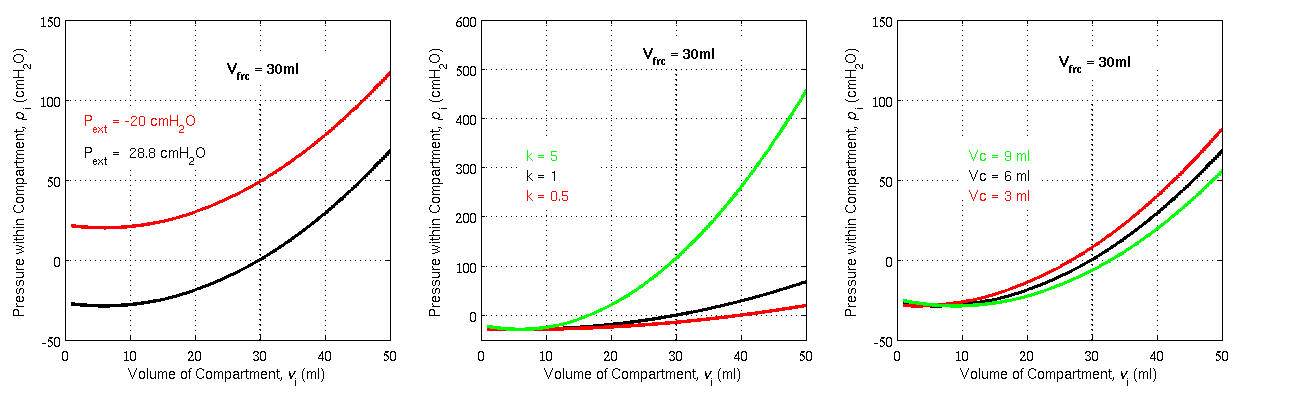 |
| --- |
| Figure S2: The effect of varying the parameters of Equation [18] on the pressure volume relationship of the model. |

For a healthy lung at the end of the expiration, the ventilator pressure would return to zero above atmospheric (resulting in the tracheal pressure also being equal to zero). The nominal values for ($P_{ext,i}$ , $S_{i}$ and$V_{c}$, see Figure S1) have been determined such that at the end of expiration, the alveolar pressure within the compartment is also equal to zero, i.e. at 30 ml, the individual compartments are at rest and consequently the total resting volume of the lung is 3 liters.

We consider each of the three parameters mentioned above ($P_{ext,i}$,$S_{i}$ and$V_{c}$) to be different yet essential components for representing a diseased lung, that affect the volume pressure relationship of the alveolar compartments. For example, for a given volume$v_{i}$, increasing$S_{i}$ increases the corresponding alveolar pressure of the alveolar compartment. When compared to another compartment with a lower$S_{i}$, a larger pressure from the mechanical ventilator would be needed to drive air into the compartment; thus effectively the compartment will be behaving as a stiffer lung unit.

Decreasing $P_{ext,i}$ increases the alveolar pressure such that the pressure gradient (especially during exhaling) forces the air out of the alveolar compartment until the volume of the compartment collapses ($v_{i}$ = 0 ml). Note that, in effect, the parameters are influencing the resting volume of the compartments (when the alveolar pressure,$p_{i}$, is equal to zero). If $p_{i}$< 0 cmH_2_O, the pressure gradient will cause the flow into the alveolar compartment (as ventilator pressure will always be ≥ 0 cmH_2_O) until $p_{i}$ reaches 0 cmH_2_O.

In the model, the total airway resistance $R_{aw}$is determined by the following equation for $N_{A}$ parallel compartments:

| $\frac{1}{R_{aw}}=\frac{1}{R_{B,1}}+\frac{1}{R_{B,2}}+\cdots+\frac{1}{R_{B,N_{A}}}, \text{for} i=1, \ldots,N_{A}$ | [19] |
| --- | --- |

where $R_{B,i}$is the bronchial inlet resistance of the $i^{th}$ compartment, which is defined by:

$R_{B,i}(t_{k})=\delta_{Bi}R_{B0}m_{i}(t_{k})$ [20]

and $R_{B0}$ corresponds to the default bronchial inlet resistance of an alveolar compartment. $R_{B0}$ is set to $1\times{10}^{-5}\cdot N_{A}$ kpa ml^-1^ min^-1^ (6 cmH_2_O l^-1^ sec^-1^); the inlet resistance is higher for a model with more compartments as the volume of each compartment decreases; giving a resistance of 0.001 kpa ml^-1^ min^-1^ for each of the 100 compartments. The coefficient$\delta_{\mathrm{Bi}}$ can be assigned for each compartment based on the patient data. $m_{i}$ is a volume dependant multiplier of the airway resistance, representing a dynamic change in airway resistance and is determined by the equation:

$m_{i}(t_{k})=1+0.1\left( \frac{V_{FRC}}{v_{i}(t_{k})N_{A}}-1 \right)$ [21]

where $v_{i,t}$ is the volume of the $i^{th}$ alveolar compartment at *t*. Additionally, a threshold opening pressure ($TOP$) at low lung volumes needs to be attained for a collapsed alveolar unit to open. To incorporate the mechanism of $TOP$, $m_{i}$ is modified as:

| $m_{i}(t_{k})=\left\{ \begin{matrix} 1+0.1\left( \frac{V_{FRC}}{v_{i}(t_{k})N_{A}}-1 \right),p_{trachea}\geq TOP_{i} \\ \infty,p_{trachea}<TOP_{i} \end{matrix} \right.$ | [22] |
| --- | --- |

where $p_{trachea}$ is the pressure in the trachea and $TOP_{i}$ is a value between 5 and 60 cmH_2_O for the $i^{th}$ alveolar compartment (16). Both $N_{A}$ (the number of alveolar compartments) and $V_{FRC}$ (the volume at functional residual capacity, assumed to be 3 litres) are fixed and set by the user (i.e. they do not change during a simulation). Therefore, during a simulation, $m_{i}$, chiefly represents the relatively small changes in inlet resistance during tidal ventilation. Furthermore, $R_{B0}$and $\delta_{Bi}$are also preset and fixed, and do not change during the simulation. The only change in airway resistance which is dynamic is $m_{i}$ which is dependent on the volume$v_{i}$at time${(t}_{k})$.

Finally, the pulmonary vascular resistance PVR is determined by

| $\frac{1}{PVR}=\frac{1}{R_{V,1}}+\frac{1}{R_{V,2}}+\cdots+\frac{1}{R_{V,N_{A}}}, \text{for} i=1, \ldots,N_{A}$ | [23] |
| --- | --- |

where the resistance for each compartment $R_{V,i}$is defined as

| $R_{V,i}=\delta_{Vi}R_{V0}$ | [24] |
| --- | --- |

$R_{V0}$ is the default vascular resistance for the compartment with a value of $160\cdot N_{A}$ dynes s cm^-5^ min^-1^, and $\delta_{Vi}$ is a coefficient that can be used to increase the vascular resistance to represent the ARDS state.

**SECTION B: Optimization Algorithms**

Genetic Algorithms have become a popular, robust search, and optimisation technique for problems with large as well as small parameter search spaces, and have been applied to a range of different problems in physiological modelling in recent years (17-19). This approach assumes that the evolutionary process observed in nature can be simulated on a computer to generate a population of fittest candidates. In a genetic search technique, a randomly sourced population of candidates undergoes a repetitive evolutionary process of reproduction through selection for mating according to a fitness function, and recombination via crossover with mutation. A complete repetitive sequence of these genetic operations is called a generation. To use this evolutionary method, it is necessary to have a method of encoding the candidate as an artificial chromosome as well as a means of discriminating between the fitness of candidates. A fitness function is defined to assign a performance index to each candidate-this function is specific to the problem and is formed from the knowledge domain.

Due to their stochastic nature, global optimisation schemes such as GA can be expected to have a much better chance of converging to a global optimal, although the price to be paid for this improved performance is a significant increase in computation time when compared with local methods. The genetic operators employed to generate and handle the population in the GA for the current problem are described below. The reader is referred to Ref. (20) for more details of different operators, binary coding schemes, and the theory of genetic search.

1. Selection: The manner in which the candidates in the current iteration (generation) are qualified for producing the successive generations depends on the selection scheme. There are many different selection schemes available (20). In this analysis, the roulette wheel selection scheme is used. Parents are selected according to their fitness. The fitter the chromosomes are, the more chance they have to be selected. This is analogous to a roulette wheel containing all the chromosomes in the population. The size of a section in the roulette wheel is proportional to the value of the fitness function of each chromosome. The selection depends on the probability factor of selection which is assigned a value 0.6 in this study.

2. Crossover: This is a recombination operator that ensures the mixing up of the information content in two binary-coded chromosomes. Usually, two parent chromosomes are selected randomly to interchange the information content, and thereby produce new off-springs that contain information content from both the parents. A probability of crossover is defined, which determines the maximum allowed number of pairs for crossover operation. In general, the probability of crossover is kept high. A simple single-point crossover scheme is employed in this study with a probability of 0.8. The information between the parents is exchanged at a randomly chosen crossover point over the length of bits.

3. Mutation: This introduces random variations in the population over the search space, by randomly flipping a bit value in the case of binary coded GA. In this study, the operation is done with a very low probability of 0.005. A binary uniform mutation scheme is used, which randomly selects an individual and sets it to a random value by flipping a randomly selected single bit.

4. Replacement strategy: An elitist strategy is followed such that over each generation the best candidate in the current population moves into the new generation population by replacing the worst candidate of the new population. This ensures the presence of a better candidate in the new generation and thereby increases the average fitness of the population over generations.

5. Termination criterion: Many different termination criteria can be employed. In the present study, an adaptive termination criterion is used that is dependent on improvement in the solution accuracy over a finite number of successive generations. The algorithm terminates the search if there is no improvement on the best solution achieved (above a defined accuracy level) for a specified minimum number of successive generations.

To speed up the optimization process, a parallelised computer code implementation of a genetic algorithm was employed in this study. The cost function evaluation process associated with a population can be accelerated hugely by distributing the tasks to multiprocessors (multiple cores and/or multiple machines). High performance computing facilities available at the University of Warwick were configured and implemented to run the parallel computing jobs.

The matching process was performed under Matlab 2015a using the Global Optimization Toolbox and Parallel Computing Toolbox. An adaptive termination strategy, which allows the optimization algorithm to run as long as necessary, was applied for each case to ensure the global optimal was reached.

The optimization problem is formulated to find the configuration of model parameters that minimize the cost function J given below:

$$\min_{x} J=\sqrt{\sum_{i=1}^{5} \frac{\hat{Y}_{i}-Y_{i}}{Y_{i}}}$$

with

$$Y=[P_{a}O_{2}, P_{a}CO_{2}, DS, VQ_{1}, VQ_{2}]$$

where $Y$ is the vector of data values and $\hat{Y}$ is the vector of model estimated values for arterial partial pressures of oxygen and carbon dioxide (P_a_O_2_ and P_a_CO_2_ respectively), dead space (DS), 0.001<V/Q<0.1 (VQ_1_) and 0.1<V/Q<10 (VQ_2_).

**SECTION C: Robustness Analysis**

Figure S3: Patient 1 - The Results of the simulations for 100 random parameter sets within ±5% of the best fit. PaCO2: partial pressure of oxygen; PaCO2: partial pressure of carbon dioxide.

Figure S4: Patient 2 - The Results of the simulations for 100 random parameter sets within ±5% of the best fit. PaCO2: partial pressure of oxygen; PaCO2: partial pressure of carbon dioxide.

Figure S5: Patient 3 - The Results of the simulations for 100 random parameter sets within ±5% of the best fit. PaCO2: partial pressure of oxygen; PaCO2: partial pressure of carbon dioxide.

**References**

1. Hardman J, Aitkenhead A. Estimation of alveolar deadspace fraction using arterial and end-tidal CO2: a factor analysis using a physiological simulation. Anaesthesia and intensive care 1999;27(5):452.

2. Hardman J, Bedforth N. Estimating venous admixture using a physiological simulator. British journal of anaesthesia 1999;82(3):346-349.

3. Hardman J, Bedforth N, Ahmed A, et al. A physiology simulator: validation of its respiratory components and its ability to predict the patient's response to changes in mechanical ventilation. British journal of anaesthesia 1998;81(3):327-332.

4. Hardman JG, Aitkenhead AR. Validation of an original mathematical model of CO2 elimination and dead space ventilation. Anesthesia & Analgesia 2003;97(6):1840-1845.

5. Hardman J, Wills J. The development of hypoxaemia during apnoea in children: a computational modelling investigation. British journal of anaesthesia 2006;97(4):564-570.

6. Das A, Gao Z, Menon P, et al. A systems engineering approach to validation of a pulmonary physiology simulator for clinical applications. Journal of The Royal Society Interface 2011;8(54):44-55.

7. McCahon R, Columb M, Mahajan R, et al. Validation and application of a high-fidelity, computational model of acute respiratory distress syndrome to the examination of the indices of oxygenation at constant lung-state. British journal of anaesthesia 2008;101(3):358-365.

8. Hardman JG, Al-Otaibi HM. Prediction of arterial oxygen tension: validation of a novel formula. American journal of respiratory and critical care medicine 2010;182(3):435-436.

9. Marshall BE, Clarke WR, Costarino AT, et al. The dose-response relationship for hypoxic pulmonary vasoconstriction. Respir Physiol 1994;96(2-3):231-247.

10. Severinghaus JW. Simple, accurate equations for human blood O2 dissociation computations. J Appl Physiol Respir Environ Exerc Physiol 1979;46(3):599-602.

11. Severinghaus JW. Blood gas calculator. J Appl Physiol 1966;21(3):1108-1116.

12. Douglas AR, Jones NL, Reed JW. Calculation of whole blood CO2 content. J Appl Physiol (1985) 1988;65(1):473-477.

13. McHardy GJ. The relationship between the differences in pressure and content of carbon dioxide in arterial and venous blood. Clin Sci 1967;32(2):299-309.

14. Kelman GR, Nunn JF. Nomograms for correction of blood Po2, Pco2, pH, and base excess for time and temperature. J Appl Physiol 1966;21(5):1484-1490.

15. Siggaard-Andersen O. The van Slyke equation. Scand J Clin Lab Invest Suppl 1977;146:15-20.

16. Crotti S, Mascheroni D, Caironi P, et al. Recruitment and derecruitment during acute respiratory failure: a clinical study. American journal of respiratory and critical care medicine 2001;164(1):131-140.

17. Anbarasi M, Anupriya E, Iyengar N. Enhanced prediction of heart disease with feature subset selection using genetic algorithm. International Journal of Engineering Science and Technology 2010;2(10):5370-5376.

18. Druckmann S, Banitt Y, Gidon A, et al. A novel multiple objective optimization framework for constraining conductance-based neuron models by experimental data. Frontiers in neuroscience 2007;1(1):7.

19. Ho W-H, Chang C-S. Genetic-algorithm-based artificial neural network modeling for platelet transfusion requirements on acute myeloblastic leukemia patients. Expert Systems with Applications 2011;38(5):6319-6323.

20. Glodberg DE. Genetic algorithms in search, optimization, and machine learning. Addion wesley 1989.
